# Lessons for Leadership Planning Employee Support in a Long-Term Crisis: The COVID-19 Healthcare Worker Experience

**DOI:** 10.1155/nrp/6417570

**Published:** 2025-09-22

**Authors:** Ruth A. Bryant, Ashton Haake, Anne Murray, Laura Genzler, Catherine R. Van Son

**Affiliations:** ^1^Nursing Administration, Abbott Northwestern Hospital, Minneapolis, Minnesota, USA; ^2^Cardio Vascular ICU, Abbott Northwestern Hospital, Minneapolis, Minnesota, USA; ^3^Penny George Institute for Health and Healing, Allina Health, Minneapolis, Minnesota, USA; ^4^Abbott Northwestern Hospital, Minneapolis, Minnesota, USA; ^5^College of Nursing, Washington State University—Vancouver, Vancouver, Washington, USA

**Keywords:** COVID-19, healthcare workers, pandemic, preparedness, qualitative research, shared-governance, well-being

## Abstract

**Background:** Numerous programs were promoted during the COVID-19 pandemic to mitigate stress and provide support. Healthcare workers (HCWs) response to these support measures has not been evaluated.

**Objective/Aim:** The objective of this study was to identify and describe the HCWs well-being and support needs over the course of the pandemic and to inform disaster planning preparations.

**Methods:** In this qualitative descriptive study, five semistructured focus groups with 24 multidisciplinary HCWs were conducted between March and June 2022. Using thematic analysis, team members read each transcript to identify patterns in the phrases and apply codes. Phrases were then compressed, and themes identified.

**Results:** Three phases were identified, each with unique themes. Phase 1) “The Beginning”: themes included “hard to know what's right”, “looked like a warzone”, “we're all in it together”, “seeing patients suffer”, and “it was kind of nonstop”. Phase 2) “Tide Turned”: themes included “trying to be strong”, “angry when they had to come back”, and “wanted it both ways”. Phase 3) “Starting to Process”: themes included “I couldn't cry then”, “started therapy, had to keep living”, and “considered leaving”.

**Conclusions:** HCWs need support during and after prolonged crises to manage ongoing fears and anxieties. Leader presence during the crisis is valuable. Disaster planning through interdisciplinary shared governance is warranted to identify and develop appropriate and meaningful interventions. Resources and plans for psychological support need to be vetted by staff. Support is required in preparation for the crisis, as well as during and after the crisis.

## 1. Introduction

The COVID-19 pandemic propelled healthcare workers (HCWs) into a state of persistent stress and anxiety that was inescapable. HCWs are not trained to manage a constant flow of mass casualties in their units, nor have they been prepared for the continuous stressors at work and home [1].

Many COVID-19 studies reported on the psychological distress experienced by HCWs, including fears of becoming infected, infecting family members, frustration, post-traumatic stress, disruption in lifestyle and routine activities, and loneliness [2–6]. These studies were survey-based and focused on the experiences of nurses and physicians in high-risk areas (i.e., critical care and emergency care) to the exclusion of other HCWs who were also impacted by the pandemic.

Typical approaches used to reduce stress pre–COVID-19 were made available during the COVID-19 pandemic, including employee assistance programs, meditation, mindfulness, helplines, yoga, exercise, and/or resilience training to enhance coping with the adverse effects of stress [7]. These practical and well-studied prepandemic resources, however, may not yield the same benefit during and after the pandemic, notably a pandemic that has been so pervasive as to impact all aspects of an HCW's life [2, 8, 9]. Additional resources were launched by management, many of which were implemented without the opportunity for HCWs to express their needs [9], and the utilization of these resources by HCWs was poor. Thus, while the psychological harm and mental health impact of COVID-19 on HCWs has been reported, it has been limited to the staff in critical care and emergency departments. In addition, the efficacy of interventions provided during a long-term crisis in an attempt to mitigate stress that was touching every aspect of the HCW's life has not been studied [8].

This study aimed to identify and describe HCWs' well-being and support needs, both met and unmet, during the COVID-19 pandemic, which can inform and improve disaster planning preparations. Our research question was: What are HCWs' attitudes and perceptions of their well-being and needs throughout the COVID-19 pandemic and their satisfaction with existing resources?

## 2. Methods

### 2.1. Study Design

The lack of pandemic research with diverse HCWs required an exploratory qualitative descriptive method to identify the breadth of the experiences related to this unprecedented event. This qualitative descriptive study collected data from focus groups between March and June 2022.

### 2.2. Participants

Purposive sampling was used to assemble an interdisciplinary group of HCWs including bedside nurses, nursing assistants, nursing leaders, pharmacists, rehabilitation therapists, providers, respiratory therapists, and ancillary workers employed at a single large healthcare center who had worked in direct patient care for at least three months between April 1, 2020, and December 31, 2020, during the COVID-19 pandemic. Inclusion criteria were 18 years of age or older, working a minimum of 24 h during a 7-day week, English-speaking, able to attend virtual meetings, and committing to at least two sessions. A flyer describing the study and inviting participation was circulated to leaders to share with their teams, using the routine hospital communication channels (bulletin boards, tier 1 and tier 2 huddles, and emails). The flyer provided a QR code to access an Intake Survey (administered through REDCap) which captured initial information to verify their eligibility, basic demographic data, the area/unit in which they worked, job title, and preferred focus group sessions. To avoid the potential for coercion or intimidation, team leaders and staff from the same unit or department were assigned to different focus groups. In addition, any co-investigator in a leadership position did not participate in focus groups and had access only to de-identified transcripts.

### 2.3. Data Collection

Five focus groups met twice over 3 months using the Zoom video conferencing platform; each focus group was video-recorded. One researcher (RAB) conducted each focus group, and another investigator took field notes. A semistructured interview guide with open-ended questions was used. The study's purpose, anonymity, pseudonym selection, and handling of the transcribed files were reviewed with participants at the beginning of each focus group. The first set of focus groups was transcribed and analyzed before conducting the second round, allowing for clarification, verification, and building on the initial data.

### 2.4. Data Analysis

Thematic analysis was used to analyze the data and organize it into themes. Research team members read each focus group transcript independently, multiple times, highlighting key phrases and concepts in the transcripts. Team members then identified patterns in the phrases and coded them accordingly. The codes were grouped to describe participant meanings and the significance of the data identified. Investigators then compared key phrases, including related significant concepts. Phrases were then compressed, and themes identified [10, 11]. Saturation was determined by the team when no new themes were identified. Study team members met weekly for 5 months.

### 2.5. Rigor

Credibility, transferability, dependability, and confirmability are paramount to attaining rigor and trustworthiness in a qualitative study [12]. The research team adhered to the qualitative research checklist (COREQ) to ensure confirmability [13]. Credibility was obtained by being transparent with participants, enlisting the Ph.D. prepared nurse scientist (RAB) to conduct the focus groups, and referencing direct quotes to support themes and subthemes. The nurse scientist did not have familiarity with the participants. Transferability was supported by inviting all disciplines that were direct caregivers during the pandemic to participate in this study, providing rich descriptions of the focus group procedures, and including pertinent quotes from the participants. An audit trail of the participant selection, focus group transcripts, and selected quotes supports evidence of dependability and confirmability.

### 2.6. Ethical Considerations

Participants were provided a consent script via email to review and encouraged to contact the research team with any questions. The consent script was then reviewed before the first focus group, and participants verbalized their understanding before initiating the discussion. Each participant received a $20 gift card for their participation.

Participant names and disciplines were not shared during the focus groups. Each selected a pseudonym at the beginning of their first focus group, which was then inserted into the electronic virtual meeting platform so that it would appear on the screen. Participants were instructed to use pseudonyms during the discussion and to keep any names confidential. Audio recordings only were uploaded to a HIPAA-compliant transcription service, and transcribed verbatim. Data were stored on an institutional network on a password-protected computer with access restricted to the study investigators. A master code list of pseudonyms and participant names was maintained in a restricted folder, separate from any data.

The Allina Health Institutional Review Board reviewed the proposal and determined it to be exempt from IRB review (# 1793046-1).

## 3. Results

### 3.1. Participant Characteristics

Twenty-four participants joined the focus groups, representing nine professions including medicine, pharmacy, dietary, transport aide, respiratory therapy, nursing assistant, and nursing (Table 1). Participants reflected a broad range of job responsibilities. Seven identified as male and seventeen as female. The participants had worked in the facility between 2 and 41 years.

**Figure 1:**
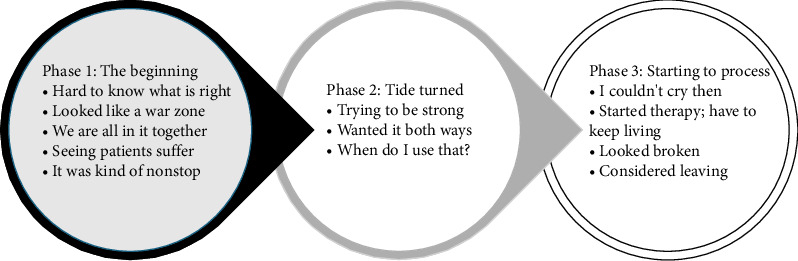
Three phases to the healthcare worker's experience during the pandemic.

Three phases of the pandemic were identified: (1) *The Beginning*, (2) *The Tide Turned*, and (3) *Starting to Process* (Figure 1). In each phase, participants identified their challenges, and the support resources used, or wished for.

### 3.2. Phase 1: The Beginning

The beginning of COVID-19 was sudden and marked by uncertainty. Every aspect of frontline HCWs' jobs changed, and information was amended daily, often hourly. Messages were mixed and usual care and protocols were replaced with less “tried and true” approaches. These new approaches were seen as incongruent with best practices and established protocols. Five subthemes were identified in Phase 1.

#### 3.2.1. Phase 1, Theme 1: “Hard to Know What Is Right”

Initially, the challenges faced by HCWs were related to knowledge and supplies. Immediately, it became apparent that this was a highly contagious communicable disease, making personal protective equipment (PPE) essential. However, the supply of PPE needed was inadequate. One provider shared: “…the biggest disappointment was that there was not enough PPE …, and that was very disappointing that they did not foresee something like this happening.” [FG-C1] The lack of supplies led to continuous changes in PPE guidelines; a nurse described: “We have always been told one mask, one patient, and then saying, well, you could use it all day. You think, is this all we have, and then you just sort of do it. I mean, the first couple of days, you are angry about it, and then after that, you are just sort of resigned to doing it.” [FG-C1] Another nurse stated: “I felt like it was like fending for yourself, protecting yourself, just using your knowledge to figure out how to protect yourself.” [FG-D1].

Equally challenging was the constantly changing information; a nursing assistant shared: “The information was hard to keep up with; one person knew one thing, that one person was wearing one thing, and the next you would walk down the hallway and somebody else was wearing something else, and it is like, ‘Okay, did I miss a memo? Did I miss something? Should I be wearing more? Is this not the right thing?' … [it] was hard to … know the right thing to do.” [FG-E1] Messages from government agencies were not helping either; a provider participant stated: “There was a realization that the CDC does not have my best interest in mind. When push comes to shove, my health is secondary because [new recommendations] seemed to go against everything we were ever taught about how to stay safe, [the message was] trust us, now get to work… a very sobering moment … I cannot depend on an agency [CDC] whose job it is to oversee this. Who can I depend on that has my health in mind?” [FG-B1].

Management's messages also changed rapidly; staff were challenged to keep up and figure out how to care for themselves and their patients. A pharmacist participant explained: “Daily things changing…, not sure if we can see patients, having very little PPE,” [FG-E1] and a nurse expressed: “feeling very helpless and like there was so much that we could do, but [no] avenue [resources] to do it.” [FG-A2] The organization's standard operating procedures and resources struggled to meet the volume and complexity of patient and staff needs during this crisis.

#### 3.2.2. Phase 1, Theme 2: “Looked Like a War Zone”

Staff were bombarded daily with procedural changes, and units operated on limited information regarding illness, transmission, care protocols, and safety. Several nursing participants described the pandemic's early months as “… very scary. Did not know really how to get it [COVID-19], and we could go from … having PPE to not having PPE, using one N95 mask for an encounter to having seven masks that we need to rotate and keep for a month…a scary time, I am expected to go into these COVID-19 rooms, and not know if I was going to get sick. Furthermore, I am seeing people die.” [FG-A1; FG-C1].

Staff were fighting an ongoing battle with an invisible invader, likening the pandemic experience to a war zone. A nurse shared: “Our unit went from a normal ICU to changing into kind of what looked like a war zone trying to figure out how we would be safe and maintain patients, and … [figuring out] how do you move a dead body out of the ICU [when] we do not know what the level of infection it was.” [FG-A1].

Informational resources failed to keep pace with the needs of HCWs to manage patient care. The trauma of tending to the onslaught of critically sick and contagious patients without essential resources stretched staff beyond their typical work-related stress thresholds. Staff were experiencing unrelenting stress with insufficient support.

#### 3.2.3. Phase 1, Theme 3: “We Are All in It Together”

In the beginning, staff capitalized on work relationships to obtain needed support. With nonessential employees now working at home, coworkers considered essential to the workplace became their source of support. One nurse said: “I felt so isolated, and no one except your coworkers truly understood what was happening.” [FG-A2] Another nurse shared: “In the beginning, we needed people to hand us supplies,… run for supplies,… and give us a break so we could run out and get a drink. It was hard not having water in your area to drink. You had to wait until someone could help so that you could go potty. It strengthened friendships and relationships.” [FG-A1].

Since information changed often, staff found that they could trust the people they worked with, so co-workers' support counterbalanced the ongoing fear of the unknown. A provider participant shared: “[The] fear of the unknown …was balanced by working with the people you trust who would help you, and that teamwork was so evident. People are looking out for one another… we are all in it together, and if the place is going to burn up, I guess we are all going down, but it was fine because you were with the people you trusted. I relied on my coworkers; we became a family,… and supported each other. I did find the camaraderie between us to be very helpful.” [FG-C1].

Organizations were juggling nonessential employees and maintaining management functions. Various strategies intended to preserve PPE and reduce viral exposures meant that leadership was not consistently physically present, leaving staff feeling less supported. When managers were physically present, providing staff support was challenging due to personnel shortages and the intensity of patient care. These conditions left staff feeling they were working without the psychosocial support unit managers typically provide. A nurse shared that some managers tried to support their staff and stated: “For the first eight or nine months … my manager held frequent daily huddles, which were super helpful [since] things changed so fast.” [FG-D1].

#### 3.2.4. Phase 1, Theme 4: “Seeing Patients Suffer”

Coupled with limited management support were the difficulties of caring for extremely sick patients. A participant illustrated the impact of the unrelenting needs of fragile patients without the resources to help them and recalled: “… seeing patients suffer, not being able to talk to their beloved ones, hug them…the isolation was too much. You see the suffering in some patients like the elderly, and they are confused, and nobody can talk to them.” [FG-E1].

The energy needed to navigate HCWs' personal needs while simultaneously experiencing the increasing volume of patients and increased severity of illness was overwhelming. A nursing assistant explained: “It was exhausting you could not take care of yourself because you did not have the support or any of the resources you normally do …, and you had more [patients] coming at you all the time.” [FG-D1].

The early weeks were demanding, but the support from coworkers and the community was ever-present. Staff tried to use existing organizational and personal resources to navigate this experience. Organizations offered additional tools and resources such as ‘coping' smartphone apps, extra pay incentives, and vouchers for grocery delivery, massage, acupuncture, etc.

#### 3.2.5. Phase 1, Theme 5: “It Was Kind of Nonstop”

Staff lived in a state of constant anxiety; no matter where they turned, they were navigating the unrelenting exposure to the virus at work and then trying to navigate life at home. A nurse shared: “…trying to juggle going to work and juggling what that looked like, juggling what school looked like, and what home looked like—it was kind of nonstop, the stress of figuring out what life looked like.” [FG-E1].

The usual coping mechanisms personnel used to separate one's workday stress and anxiety from home life became ineffective during the pandemic. A nurse revealed: “…different than anything I have experienced in my career is that when I go home, I could separate that anxiety, but with COVID-19, you went home, and it was on the news, and it was on your social media feeds, and it was everywhere…so, lots of anxiety.” [FG-C1].

### 3.3. Phase 2: “Tide Turned”

Participants noted a change within the community and the patients they served when COVID-19 testing was more accessible and vaccines became available. Support from the community waned, and patients were not taking advantage of developing resources to protect them from contracting the virus. This situation was difficult for HCWs to cope with, and pandemic fatigue and stressors increased mental health distress. Three themes were identified in Phase 2.

#### 3.3.1. Phase 2, Theme 1: “Trying to Be Strong”

As time wore on, the pandemic took its toll. A nurse assistant shared: “Nobody is acting as they normally would, so then they [patients] are taking it out on us; we are already stretched to the max…I felt like I had everything pouring down on me…but I was trying to be strong. I was emotionally exhausted. I knew I was needed and kept pushing myself more… I was picking up shifts trying to help out.” [FG-D1].

#### 3.3.2. Phase 2, Theme 2: “Wanted It Both Ways”

As the months wore on, support began to waver from organizational management and the community. Community members hesitated about whether to wear a mask or obtain vaccinations. Staff found it challenging to remain altruistic when caring for patients who were not taking advantage of preventive measures and demanding resources that were in short supply, while exposing HCWs to the virus. A nursing assistant shared: “…HCWs have always been so well respected,… and initially, that was the case, and then it seemed to be like a tide turned at some point where they [the public] were not taking any precautions, and they got mad when we asked them to wear a mask or be vaccinated.” [FG-E1] A physician expressed frustration with patients who “… came in and had not been vaccinated [and] wanted it both ways. They did not want to be vaccinated because that is my right, but when I get sick, I still want to take up a bed in the hospital. It was challenging to understand how there were people who did not see the dangers of the virus, do nothing to protect themselves, and still expect care… It felt so severe to me as a [physician] that everyone was like, ‘Who cares, deal with it? If I get sick and I come in, you must take care of me, so deal with it.' I was angry and disappointed.” [FG-E1].

#### 3.3.3. Phase 2, Theme 3: “When Do I Use That?”

Organizational messages called for top-level job performance regardless of the level of support offered at any given time. Although masks and vaccines were now available, there continued to be a staffing shortage in all areas. As the pandemic continued, staff support, which had flourished in the beginning, waned. Staff were aware of support still being offered within other organizations and, at times, questioned the nature and extent of support they were receiving from their own. Staff found it hard to schedule time with resources such as the Employee Assistance Program. New resources such as massage therapists, acupuncturists, and smartphone apps, were offered but were not as useful as intended. A nurse explained: “Staffing is just an ongoing problem; I am still working 40-plus hours… at what point do we take care of ourselves? You can bring an acupuncturist in every Tuesday from 11:00 to 1:00, but when do I get to use that? Because I am on the floor [and cannot take] a break.” [FG-E1] A physician discussed exploring one of the new smartphone apps that was made available. She said: “Doing the apps sometimes was more exhausting; it required reading or mental focus, and I just didn't have the capacity to do that. I dug into one of the apps …[its assessment was] ‘We have identified you are depressed.' And I was like, I know that!” FG-E1].

### 3.4. Phase 3: “Starting to Process”

Ultimately, HCWs began questioning career decisions; the struggles and sacrifices made after 2 years were too much. Some participants spoke about trying to process what they had experienced, and others described this time as secondary trauma. Experiencing so many patient deaths, completely isolating patients from close family and friends, and even denying patients human touch depleted all the psychological resources one had. Four themes were identified in Phase 3.

#### 3.4.1. Phase 3, Theme 1: “I Couldn't Cry Then”

HCWs put their psychological needs on hold and functioned moment by moment, day by day. Thus, processing their feelings and experiences was suspended. A nurse described her experience: “I am just starting to process what I went through, the number of deaths I saw; I couldn't cry then. I had no one asking me how I felt… I couldn't cry for my patients. I could be there as they died and hold their hand…I'd give them the phone and let them call their family before they were intubated, I'm a caring person and I did my best for them, and I couldn't cry for them. But it's starting to come out now.” [FG-C1].

#### 3.4.2. Phase 3, Theme 2: “Started Therapy, Have to Keep Living”

Participants spoke of seeking professional help outside of work to move forward. Some believed that their experience during COVID-19 led to having post-traumatic stress disorder (PTSD). One of the HCWs shared the following: “I started therapy for the first time in my life… and I am realizing, in the last few months how much it affected me. I did a good job at shoving it down or aside because you must keep going to work and living. I still do not fully realize how much it affected me.” [FG-B2] Another HCW put it this way: “I'm starting to climb out of a hole…there was much damage done in these last 2 years, and I'm just trying to recover and take care of myself.” [FG-D1].

#### 3.4.3. Phase 3, Theme 3: “Looked Broken”

In addition to their own experiences, HCWs witnessed their coworkers' experiences, magnifying their trauma. A pharmacist shared his observation: “Some nurses …looked broken like they are traumatized. One nurse shared with me, ‘I'm seeing a lot of young women, like in the early twenties, who are pregnant and not vaccinated, and they are dying. And often their babies are too.' And he looked broken from that. He was not crying as he told us, but you could feel the weight he was carrying.” [FG-E1].

#### 3.4.4. Phase 3, Theme 4: “Considered Leaving”

For some, the unrelenting stress of working in a healthcare setting with COVID-19 patients and trying to maintain family life and engage in community activities led to pure exhaustion and burnout. A nurse shared: “On that exhaustion note, I considered quitting, I considered leaving. I went down to 2 days a week. It is the least I can work [and] still have benefits.” [FG-E1] While a nursing assistant expressed: “I realized I am burnt out, I can't do this … reached a point where [I think] what have I sacrificed? I have been a bad parent for a year. My relationships are stressed. I am stressed, [and] for what?” [FG-C1] The pandemic led to an exodus of frontline HCWs. They were not prepared to manage the hardships of a crisis experienced across the globe.

## 4. Discussion

The nurse's experience caring for patients during the COVID-19 pandemic, particularly in the ICU, has been described [14, 15]. In contrast, this study included a variety of disciplines, described their experiences of the pandemic over time, and explored what resources were beneficial. This exploration is valuable because an organization's resilience and adaptive ability are partly measured by its responses to various crises [16].

Results from this study highlighted that while engaged in the usual time-limited disaster planning, the scope of any plan must include sufficient HCW-valued psychosocial support. In addition, the presence of leaders, management, and key team members (such as pharmacists, social workers, and chaplains) during the crisis is crucial in managing feelings of vulnerability in frontline HCWs.

HCWs in this study felt ill-prepared for the ongoing difficulties presented by this pandemic. While well intended, the numerous resources that were made available to staff (e.g., employee assistance programs, apps for food delivery services or resilience training, counseling, massages, and acupuncture) were perceived as not helpful. Staff were frustrated because the ability to access these resources was hampered by being overwhelmed, exhausted, and lacking time; they were left with a perception that the organization did not understand their needs.

While war-related language to describe the pandemic is discouraged [17], lessons can be learned and applied from the military experience in terms of (1) disaster preparedness during noncrisis times, (2) how to prepare HCWs for a disaster at a moment's notice, and (3) preparing for long-term engagement [18]. Disaster planning with input from diverse HCWs is needed to develop and identify meaningful resources and programs that will meet the challenges during and beyond the acute phase of any public health disaster [19, 20]. Such planning can occur through an interdisciplinary shared governance structure, rather than the typical vertical model of management. This type of structure provides an opportunity for all HCWs (nurses, providers, therapists, dietary, etc.) to collaboratively make informed decisions about their work environment [21]. Operationalizing an interdisciplinary shared governance requires a culture that supports collaboration, mutual trust, respect, and leaders who can mentor and facilitate as needed [22]. Such reconfiguring of disaster planning and staff resources may also strengthen HCW resilience over time [16]. Because experiences varied across disciplines, an interdisciplinary shared governance structure also provides a mechanism for HCWs to identify and implement support resources that reflect the unique needs of each discipline.

Participants described feelings and experiences that mirrored findings in other studies, in particular identifying signs and symptoms of PTSD [23, 24]. A higher level of preparedness, attention to workload and rest, recognition of efforts, and opportunities to share experiences with colleagues can reduce levels of post-traumatic stress in times of crisis [25, 26].

## 5. Strengths and Limitations

The strength of this study is that it reports on the experiences of a multidisciplinary group of HCWs rather than being limited to nurses or providers. A limitation of this study is that it was conducted in a single acute care institution and had a small number of participants. While two potential limitations included conducting the focus group interviews virtually using a videoconferencing format and including a mix of disciplines in the focus groups, these did not appear to be limitations in that participants were eager to share and contribute to the discussion. In addition, the teleconferencing format provided greater opportunity for diverse HCWs to participate. We were unable to share our findings directly with participants to enhance confirmability; however, employees have since expressed agreement with our findings following poster and oral presentations of this research at local conferences.

## 6. Conclusions

This study offers disaster preparedness guidance from the perspective of HCWs, which extends beyond the usual logistics planning. Operationalizing an interdisciplinary shared governance structure to participate in disaster preparedness training during noncrisis times should include identifying support resources that are vetted by the HCWs. In addition, disaster planning must consist of plans and resources that address the challenges beyond the usual acute phase of public health disasters. The visible presence of leaders and frequent staff contact, such as daily huddles, is essential throughout the entire crisis.

## Figures and Tables

**Table 1:** Participant characteristics.

	*N* = 24
Gender	
M	7
F	17
Profession	
Ancillary staff	1
Nurse	9
Nurse leader	1
Nursing assistant	1
Provider	4
Pharmacist	1
Rehab therapist	2
Other	5
Years in profession	
0–3	3
> 3–5	2
> 5–10	6
> 10–20	6
> 20–30	5
> 30+	2
Years employed at current site	
0–1	1
> 1–2	3
> 2–5	3
> 5–10	8
> 10–20	2
> 20–30	4
> 30	2
No response	1

*Note:* Provider = physician, physician's assistant, advanced practice nurse. Ancillary staff = dietary, transport aide, housekeeping, etc. Other = social worker, radiation therapist.

## Data Availability

The data that support the findings of this study are available on request from the corresponding author. The data are not publicly available due to privacy or ethical restrictions.
